# An improved daily standardized precipitation index dataset for mainland China from 1961 to 2018

**DOI:** 10.1038/s41597-022-01201-z

**Published:** 2022-03-30

**Authors:** Qianfeng Wang, Rongrong Zhang, Junyu Qi, Jingyu Zeng, Jianjun Wu, Wei Shui, Xiaoping Wu, Jianwei Li

**Affiliations:** 1 Fujian Provincial Key Laboratory of Remote Sensing of Soil Erosion, College of Environmental & Safety Engineering, Fuzhou University, Fuzhou, 350116 China; 2 College of Physics and Information Engineering, Fuzhou University, Fuzhou, 350116 China; 3 Joint Global Change Research Institute, Pacific Northwest National Laboratory and University of Maryland, College Park, MD 20740 USA; 4 Earth System Science Interdisciplinary Center, University of Maryland, College Park, 5825 University Research Ct, College Park, MD 20740 USA; 5 State Key Laboratory of Earth Surface Processes and Resource Ecology/Faculty of Geographical Science, Beijing Normal University, Beijing, 100875 China

**Keywords:** Climate change, Natural hazards

## Abstract

The standardized precipitation index (SPI), one of the most commonly used drought indicators, is widely used in the research areas of drought analysis and drought prediction in different fields such as meteorology, agriculture, and hydrology. However, its main disadvantage is the relatively coarse time resolution of one month. To improve the time resolution of SPI to identify flash droughts, we have refined the traditional SPI calculation method and developed a new multi-scale daily SPI dataset based on data from 484 meteorological stations in mainland China from 1961 to 2018. SPI data from three different sites (located in Henan, Yunnan, and Fujian Provinces) at the three-month timescale were analyzed by comparing with historically recorded drought events. We found that the new multi-scale daily SPI can effectively capture drought events in different periods and locations and identify the specific start and end times of drought events. In short, our SPI dataset appears reasonable and capable of facilitating drought research in different fields.

## Abstract (summary)

**Table:** 

Measurement(s)	Standardized Precipitation Index
Technology Type(s)	data transformation
Factor Type(s)	observation period • temporal resolution • spatial resolution
Sample Characteristic - Environment	drought • climate change • climate extremes
Sample Characteristic - Location	mainland China

## Background & Summary

Drought is the most frequent, complex, chronic, and severe natural disaster worldwide^1–3^. Drought areas caused by water deficits have significantly spread in the past several decades across China because of climate change^4,5^. Northwestern China suffers from severe water resource crises and drought risk^6^. Drought can lead to adverse effects on drinking water, water resource availability, agricultural production and yield, and ecological environment and ecosystem stability^7–9^. Therefore, drought monitoring and evaluation have become hot topics of discussion, attracting the attention of hydrologists, ecologists, geographers, meteorologists, and non-scientists^10,11^. There is evidence that extreme climate events, including droughts, will be intensifying in this century in spatiotemporal terms under climate change^12–16^. However, assessments of the evolution and spatiotemporal characteristics of drought resulting from water anomalies at the country scale are lacking^1,2^. It is thus imperative to evaluate, monitor, and assess drought characteristics using long time-series data at a large scale, which can play important roles in water resources management, responses to alleviating drought, and drought risk management.

The American Meteorological Society considers different drought definitions and divides droughts into four main categories, i.e., meteorological drought, agricultural (soil moisture) drought, hydrological drought, and socioeconomic drought^17,18^. Drought indices have been developed as effective tools to monitor and evaluate the spatiotemporal characteristics of different drought types^1,19^. The three most popular and representative drought indices are the standardized precipitation index (SPI)^20^, the Palmer drought-severity index^21^, and the standardized precipitation evapotranspiration index (SPEI)^22^. Other widely used indices include the surface water supply index^23^, the evaporative demand drought index^24^, and the vegetation condition index^25^, among others. SPI and SPEI have multi-time-scale characteristics for monitoring the different drought types^22^. However, the calculation of SPEI requires the reference evapotranspiration parameter of the research areas or stations^1^, leading to different SPEI results due to different calculation methods of reference evapotranspiration using the same input data^22,26^. SPI has the advantage of a simple calculation procedure and the flexibility of different timescales^20^. It has been adopted by the World Meteorological Organization as a global tool to monitor drought characteristics^20,27^. SPI is thus an effective tool and index to monitor the different kinds of drought.

SPI standardizes the deviation from the mean of precipitation, allowing comparisons under dry (water deficit) or wet (water surplus) conditions^20^. SPI can not only be calculated simply and has spatial comparability in humid and arid zones^22,28^, but it also can obtain and recur drought events detected by other indices^29,30^. SPI can detect meteorological drought, soil moisture conditions or agricultural drought, long-term hydrological drought, and socioeconomic drought for the short accumulation of the water balance on 1-month, 3-month, 6-month, and 12-month and longer timescales, respectively^20^. Previous studies have reported the robustness and better performance of SPI compared to other drought indices^28,31,32^. Although SPI has been widely accepted and successfully used to monitor and evaluate the characteristics and risk management of the different drought types^33–36^, it is generally calculated or obtained using monthly precipitation amounts. The monthly SPI only can detect the month of onset and termination of drought^26,27^ and cannot identify the onset and termination days of drought events. It is imperative to develop SPI at a daily resolution for detailed monitoring and assessment of drought characteristics, especially flash drought.

Our primary aim is to produce and provide a daily drought index dataset with a long time series (1961–2018) at meteorological stations in mainland China so that different kinds of drought characteristics can be monitored and evaluated. The multi-time-scale SPI at three typical sites will then be used to verify the validity of the index and analyze the drought characteristics of the three-month-scale SPI in China. Our dataset is anticipated to monitor and assess the characteristics and impacts of drought to cope with climate change. It can also be used to evaluate the impact of drought on the ecosystem, crop growth, crop yield, vegetation phenology, and plant activity.

## Data and Methods

### Data sources

**Fig. 1:**
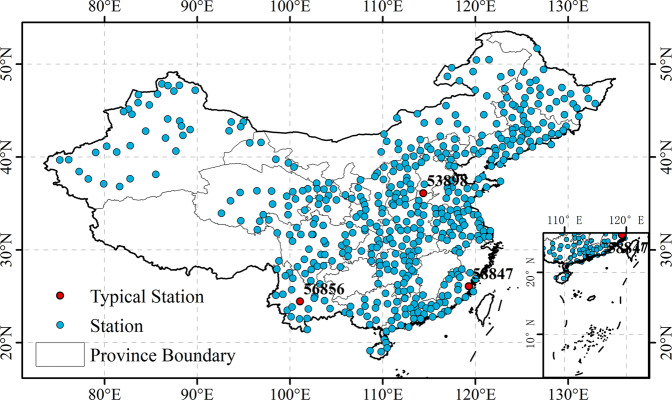
The distribution of meteorological stations across the mainland China, including three typical stations (station 53898 in the Henan, station 56856 in Yunnan, and station 58847 in Fujian).

We used daily precipitation data from 484 meteorological stations in mainland China from 1961 to 2018 provided by the China Meteorological Data Sharing Service Platform to calculate the SPI dataset (http://data.cma.cn/). These data have undergone strict quality control on the platform and have been widely used in the calculation of various drought indices and drought assessments^37^. The platform provides free meteorological data from 839 meteorological stations in mainland China. To ensure the continuity and completeness of data records, we selected precipitation data from 484 stations for calculation and analysis. Figure 1 shows the distribution of these 484 stations. Three typical sites (i.e., station numbers 53898, 56856, and 58847, marked in red in Fig. 1) are located in Henan, Yunnan, and Fujian Provinces, respectively. Among them, the Henan and Yunnan sites have experienced frequent drought disasters since ancient times^38,39^. Previous studies have reported that although the precipitation in Fujian is relatively sufficient, the distribution of precipitation is uneven, and it is also prone to drought^2,40–42^. Therefore, we take these three typical sites as examples to investigate the precipitation profit and loss monitoring capabilities of the improved daily SPI.

### Daily SPI calculation

The daily SPI is obtained by fitting and normalizing precipitation data with different probability distribution functions. Many studies have explored the effects of different probability distribution functions on the SPI calculation^43,44^. Commonly used probability distribution functions are Gamma, Weibull, and Gumbel. Among them, the Gamma distribution is the best in calculating SPI because of its relatively flexible shape parameter^45^. Before calculating the probability distribution, the cumulative precipitation time series at different timescales is needed. In this study, we used the following functions to construct the daily precipitation time series at different timescales (e.g., 30 days):

$${X}_{i,j}^{k}=\mathop{\sum }\limits_{l=31-k+j}^{30}{P}_{i-1,l}+\mathop{\sum }\limits_{l=1}^{j}{P}_{i,l},\quad {\rm{if}}\;j < k\;and$$

$${X}_{i,j}^{k}=\mathop{\sum }\limits_{l=j-k+1}^{j}{P}_{i,l},\quad {\rm{if}}\;j\ge k$$

where ${X}_{i,j}^{k}$ is the cumulative precipitation amount on a given day *j* in year *i* at timescale *k* (days), and *P*_*i,j*_ is the daily precipitation amount on day *j* in year *i*.

We then introduced the gamma probability distribution function to calculate the probability distribution of the accumulated precipitation time series. The probability density function is

$$f\left(x\right)=\frac{1}{{\beta }^{\gamma }\Gamma \left(\gamma \right)}{x}^{\gamma -1}{e}^{-\frac{x}{\beta }},\quad x > 0,$$

$$\Gamma \left(\gamma \right)={\int }_{0}^{\infty }{x}^{\gamma -1}{e}^{-x}dx,$$

where the random variable *x* is the cumulative precipitation time series at a certain timescale. *β* > 0 and *γ* > 0 are scale and shape parameters, respectively, calculated by the maximum likelihood estimation method as follows:

$$\widehat{\gamma }=\frac{1+\sqrt{1+\frac{4}{3}A}}{4A},$$

$$\widehat{\beta }=\frac{\bar{x}}{\widehat{\gamma }}$$

$$A={\rm{lg}}\bar{x}-\frac{1}{n}\mathop{\sum }\limits_{i=1}^{n}{\rm{lg}}{x}_{i},$$

where *x*_*i*_ is the cumulative precipitation time series at a certain timescale, *n* refers to the number of precipitation time series samples, and $\bar{x}$ refers to the average of the precipitation time series samples.

The probability that the random variable *x* is less than the precipitation *x*_0_ on a certain time scale is

$$P\left(x < {x}_{0}\right)={\int }_{0}^{{x}_{0}}f\left(x\right)dx.$$

Since the domain of the gamma function does not include the case of *x* = 0 and the actual precipitation amount may be 0, the piecewise probability distribution is then

$$P\left(x\right)=\left\{\begin{array}{c}{P}_{0}+\left(1-{P}_{0}\right)F\left(x\right)\quad x > 0\\ \frac{m+1}{2(n+1)}\quad \quad \quad \quad \quad x=0\end{array}\right.,$$

where *P*_0_ refers to the historical ratio of periods with zero precipitation. *F*(*x*) is the probability distribution for samples with detectable accumulated precipitation. The parameters *n* and *m* represent the number of samples and the number of samples where total precipitation equals zero, respectively.

Next, the gamma probability distribution is normalized:

$$P\left(x < {x}_{0}\right)=\frac{1}{\sqrt{2\pi }}{\int }_{0}^{{x}_{0}}{e}^{-\frac{{z}^{2}}{2}}dx.$$

Finally, SPI is obtained:

$$SPI=z=S\frac{{c}_{0}+W-{c}_{1}W-{c}_{2}{W}^{2}}{1+{d}_{1}W+{d}_{2}{W}^{2}+{d}_{3}{W}^{3}},$$

$$W=\sqrt{{\rm{ln}}\frac{1}{{P}^{2}}}\left\{\begin{array}{l}P=1-F\left(x\right),S=-1\,F\left(x\right)\le 0.5\\ P=1-P,S=1\,F\left(x\right) > 0.5\end{array}\right.,$$

where the constants are *c*_0_ = 2.515517, *c*_1_ =0.802853, *c*_2_ =0.010328, *d*_1_ = 1.432788, *d*_2_ =0.189269, and *d*_3_ = 0.001308.

Based on the commonly used monthly SPI, we developed daily SPI at different timescales (i.e., 1-month, 3-month, 6-month, 9-month, and 12-month timescales) using the method described above. Referring to the classification standard of meteorological drought in China, SPI is divided into nine categories, as shown in Table 1.

**Table 1:** Drought classification of different grades based on SPI.

Categorization	SPI values
Extremely Wet	SPI ≥ 2
Severely Wet	1.5 ≤ SPI < 2
Moderately Wet	1 ≤ SPI < 1.5
Mildly Wet	0.5 < SPI < 1
Normal	−0.5 ≤ SPI ≤ 0.5
Mild Drought	−1 < SPI < −0.5
Moderate Drought	−1.5 < SPI ≤ −1
Severe Drought	−2 < SPI ≤ −1.5
Extreme Drought	SPI ≤ −2

### Theory of runs

Based on SPI and run theory, drought characteristics were analyzed. A run in run theory is an unbroken sequence of similar events in a given ordered sequence of two or more types of symbols^46^. A drought event generally has four drought characteristics: duration, severity, intensity, and frequency^47^. The determination method of similar events is whether SPI is within the same specified threshold. Drought duration refers to the duration of a certain level of drought event from the beginning (*t*_*n*_) to the end (*t*_*p*_), i.e.,

$${\rm{Drought}}\;{\rm{duration}}={t}_{n}-{t}_{p}.$$

Drought severity refers to the sum of SPI during drought events:

$${\rm{Drought}}\;{\rm{severity}}={\sum }_{{t}_{p}}^{{t}_{n}}\left|{\rm{SPI}}\right|.$$

Drought intensity is the average value of SPI at a certain level of the drought event:

$${\rm{Drought}}\;{\rm{intensity}}=\frac{{\rm{Drought}}\;{\rm{severity}}}{{\rm{Drought}}\;{\rm{duration}}}.$$

**Fig. 2:**
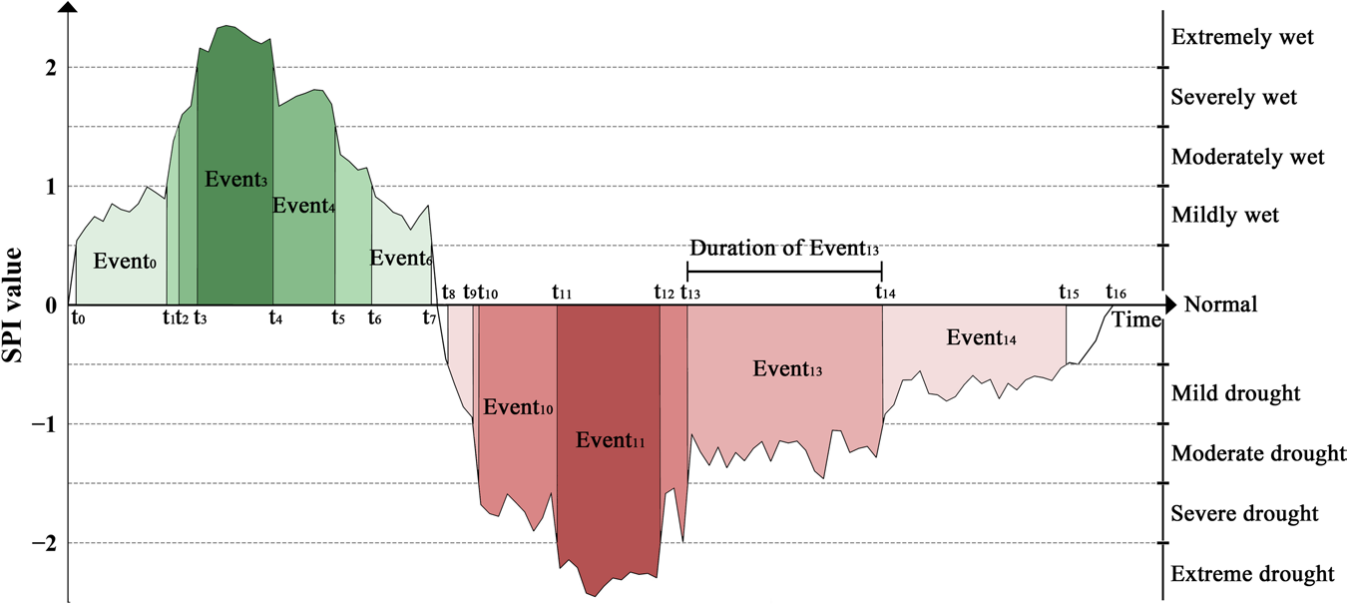
Schematic diagram of drought levels. Different colors represent different levels of drought and wet events.

The number of drought events at a certain level within a certain period is defined as the drought frequency. Figure 2 shows the definition and relationship between drought events and their attribute characteristics.

We also used data from three typical stations as examples to analyze the characteristics of drought events in different regions. As Fig. 1 shows, the three typical regional sites chosen are located in Henan, Yunnan, and Fujian Provinces (site numbers 53898, 56856, and 58847, respectively). To better compare and analyze the characteristics of drought events in China, we took the three-month scale as an example to calculate the annual total drought intensity (ATDS), annual total drought duration (ATDD), and annual total drought frequency (ATDF) at all sites^48^.

### Statistical methods

We used the Theil-Sen (TS) method to estimate the long-term trends of the ATDS, ATDD, and ATDF at all stations. The TS estimator selects the median of the slopes of all straight lines for two-dimensional sample points to estimate the trend^49,50^. It has proven to be a robust method for monitoring trends in time series and not strongly affected by outliers^51^. The Mann-Kendall method was then used to test the significance of the long-term trends of the ATDS, ATDD, and ATDF at all stations. As a nonparametric test method, the Mann-Kendall approach does not require the data to follow a normal distribution^52,53^. The TS estimator and Mann-Kendall method have been widely used in many fields, for example, in studying the water environment, ecological remote sensing, and climate change^54,55^.

## Data Records

The new daily SPI dataset developed here contains SPI values at five timescales (1-month, 3-month, 6-month, 12-month, and 24-month) from 484 weather stations in mainland China, covering the period of 1961 to 2018. All daily SPI datasets can be freely accessed through the online open-access repository called *figshare*^56^, available at 10.6084/m9.figshare.14135144. In the future, we will continue to update the SPI dataset, adding SPI data from the most recent years.

The SPI dataset contains two compressed files in the RAR format, representing two different storage methods. Either can be downloaded according to a researcher’s particular needs. The file “daily_SPI_data_multi-scale_by_each_station.rar” is stored according to the site and includes a description document “readme.txt”, a site information table “SPI_station_lookup_table.csv”, and a folder labeled “daily_SPI_data_multi-scale”. This folder includes another description document “readme.txt” and 484 XLXS files. Each XLSX file is named with a site number and contains five sheets representing the SPI values at five different event scales at the site (1-month, 3-month, 6-month, 12-month, and 24-month timescales). The file “SPI_station_lookup_table.csv” contains detailed information about each site. Similarly, the file “daily_SPI_data_multi-scale_by_each_station.rar” is stored according to different SPI timescales at the different sites. The five SPIs at different timescales are divided into five folders for storage purposes. Each folder contains 484 SPI site data files in the CSV format.

## Technical Validation

### Analysis of drought characteristics of typical stations

**Fig. 3:**
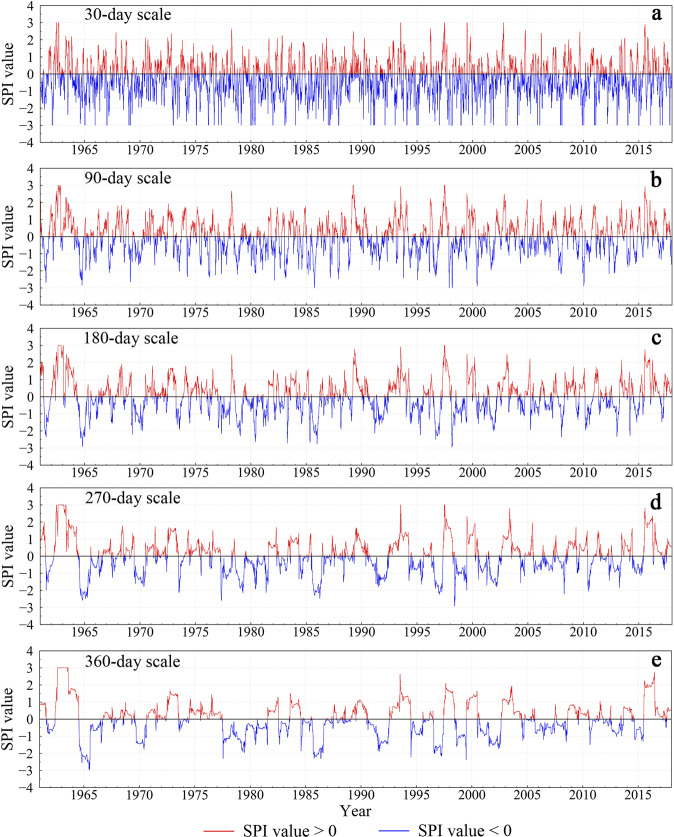
Time series of SPI at different timescales for station 53898 (Henan) from 1962 to 2018. The red and blue portions of the curves show SPI values greater than and less than zero, respectively.

**Fig. 4:**
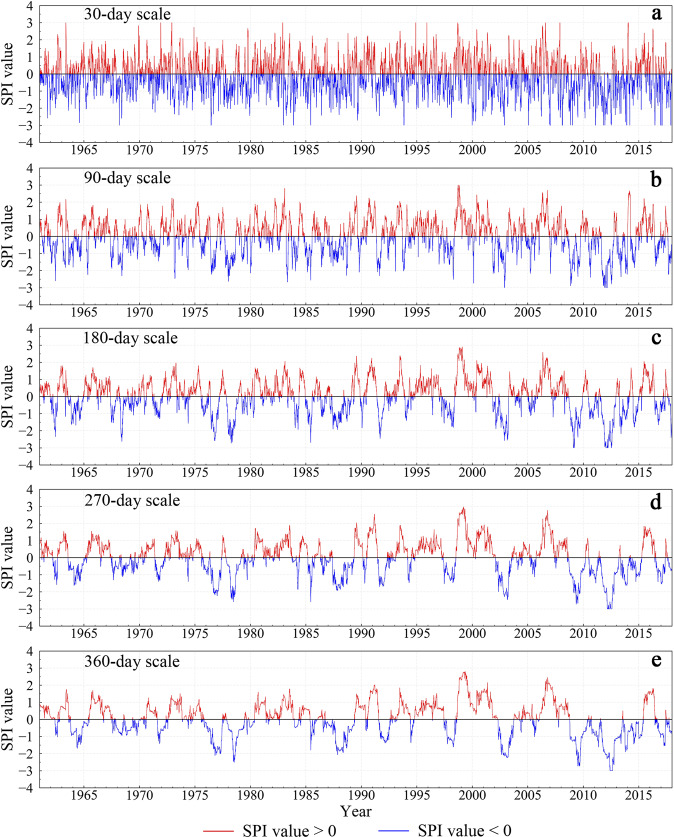
Time series of SPI at different timescales for station 56856 (Yunnan) from 1962 to 2018. The red and blue portions of the curves show SPI values greater than and less than zero, respectively.

**Fig. 5:**
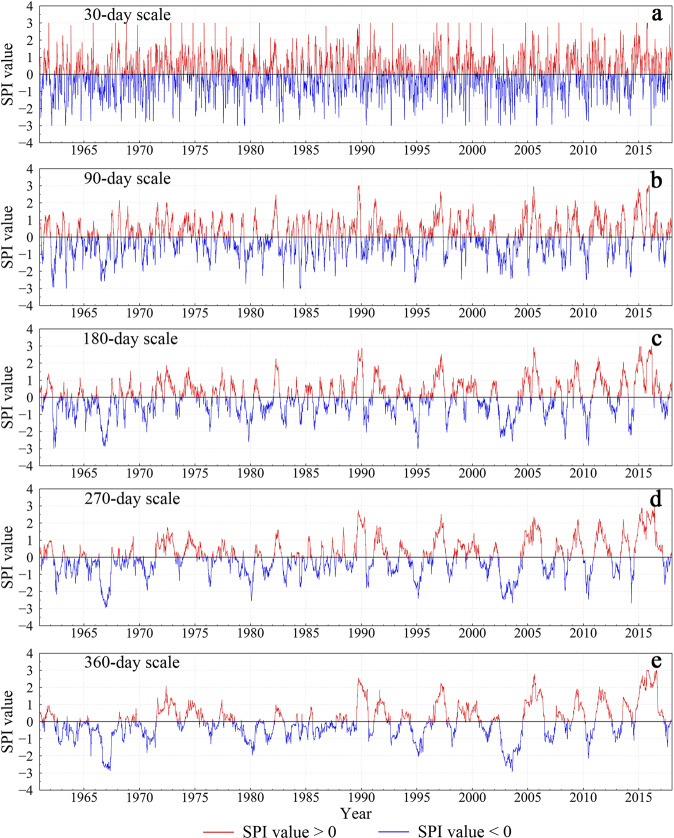
Time series of SPI at different timescales for station 58847 (Fujian) from 1962 to 2018. The red and blue portions of the curves show SPI values greater than and less than zero, respectively.

Figures 3–5 show the time series of SPI at stations 53898 (Henan), 58847 (Fujian), and 56856 (Yunnan) from 1962 to 2018 at different time scales. In general, the shorter the time scale, the more sensitive the SPI is to short-term precipitation, and the greater the range of SPI values changes. Periodic changes in the SPI value are seen at shorter timescales. Peaks in the curves are mostly concentrated during the rainy season from April to September each year.

Figure 3 shows that station 53898 (Henan) experienced severe drought disasters in 1965, 1966, 1978, 1986, 1992, and 1993, consistent with the drought disaster events recorded in *The Henan Volume of the Chinese Meteorological Dictionary*^57^. According to records, the drought disasters that occurred in Henan Province from 1965 to 1966 were extremely serious, causing rivers and wells to dry up. Precipitation in the northern part of Henan, where station 53898 is located, was reduced by more than 60%, compared with the normal annual precipitation. Figure 3 shows that the SPI value at this station reached an abnormal value of −3 at points from 1965 to 1966.

According to *The Yunnan Volume of the Chinese Dictionary of Meteorological Disasters*^58^, years of severe drought in Yunnan from 1949 to 2000 include 1963, 1987, 1988, 1992, and 1998. Figure 4 shows that unlike station 53898, extreme drought events at station 56856 in Yunnan Province mostly occurred after 2000. According to *The China Meteorological Disaster Yearbook*^59^ from 2004 to 2018, Yunnan Province experienced severe droughts from 2003 to 2004 and from 2009 to 2013, consistent with Fig. 4.

Compared with the stations in Yunnan and Henan, the station in Fujian Province had plenty of rainfall, but droughts still frequently occurred. Figure 5 shows that the SPI curve at the monthly scale was greatly affected by short-term precipitation, with no obvious drought phenomenon detected. However, the SPI curves of the 3-month, 6-month, 9-month, and 12-month timescales all showed drought conditions of differing degrees in 1963, 1977, 1971, 1970, 1980, 1983, 1986, 1991, 1995, 2003, and 2004. This is identical to the records of *The China Meteorological Disaster Dictionary*^60^ and *The China Meteorological Disaster Yearbook*^59^.

### Spatial distributions of drought characteristics

**Fig. 6:**
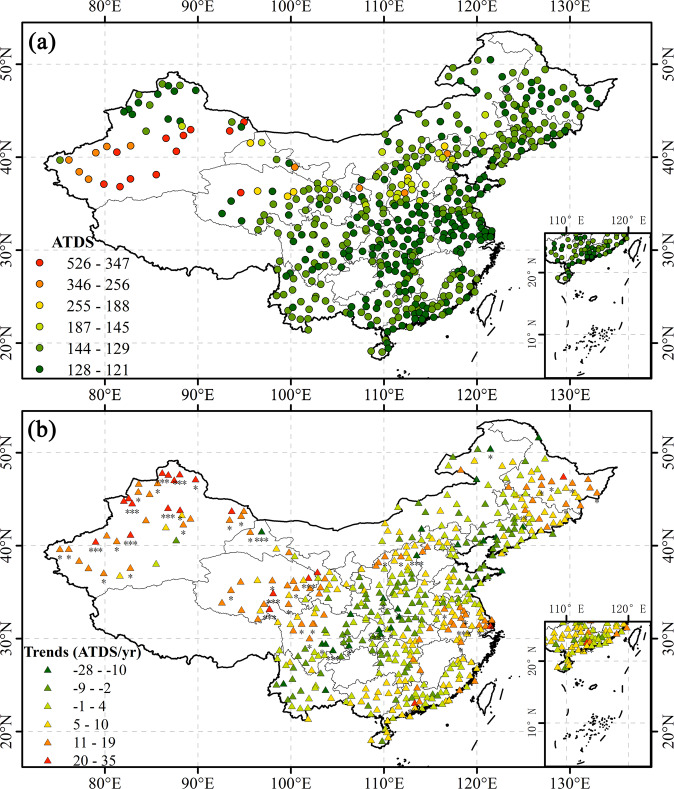
Distributions of (**a**) ATDS in the study area and (**b**) its trends at each station (“***” denotes P-value < 0.001, “**” denotes P-value < 0.01, and “*” denotes P-value < 0.05).

Figure 6 shows the spatial distributions of ATDS and its trends at 484 stations in mainland China. The lower the ATDS value, the stronger the drought severity accumulated over the years and the more severe the drought experienced at the station. ATDS values at most stations fell between −130 and −121. The Xinjiang region in northwestern China and the provinces of Hebei and Shanxi in the central part of China experienced more severe droughts, with ATDS values between −155 and −526. In general, the drought in northern China was more severe than in the south. However, compared with other areas in northern China, the drought in Heilongjiang and Jilin in the northeast was relatively mild (Fig. 6a). The multi-year trend in ATDS in the study area is not highly significant. The drought in Xinjiang, Qinghai, and other places in northwestern China showed some easement, with a trend greater than 30 (*P*-value* < *0.05; Fig. 6b).

**Fig. 7:**
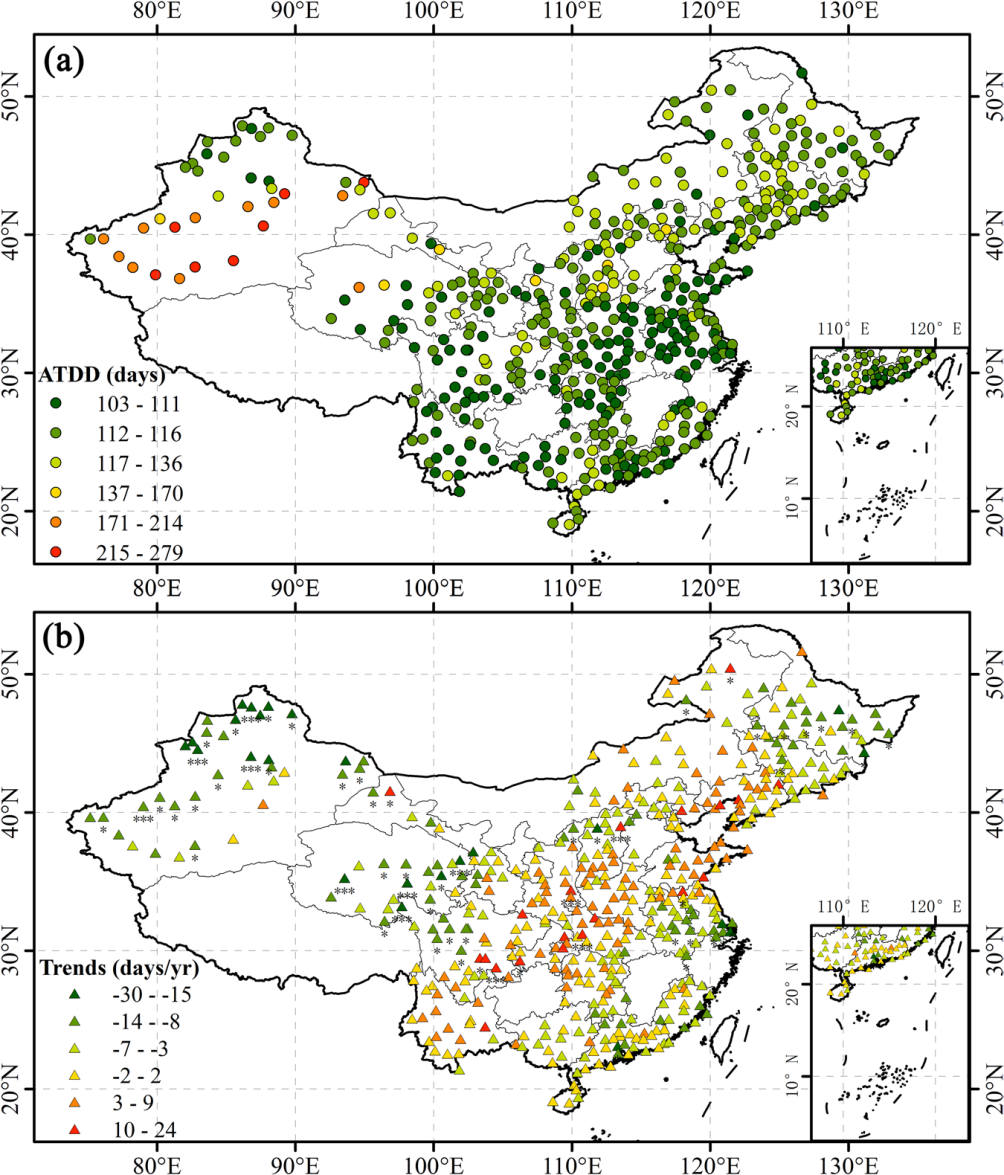
Distributions of (**a**) ATDD in the study area and (**b**) its trends at each station (“***” denotes P-value < 0.001, “**” denotes P-value < 0.01, and “*” denotes P-value < 0.05).

The variable ATDD represents the duration of the annual average drought event at each station and has similar spatial distribution characteristics as ATDS. ATDD values at some stations in the Xinjiang region of northwestern China ranged from 196 to 279. Even in southern regions with abundant rainfall, ATDD values at most stations ranged from 103 to 112, indicating that most sites experienced drought (Fig. 7a). The multi-year trend of ATDD also shows that the drought duration at some stations in the southwest, southeast, and northeast regions of China had significantly reduced, while the drought duration at some stations in central and southwestern regions had increased significantly (Fig. 7b).

**Fig. 8:**
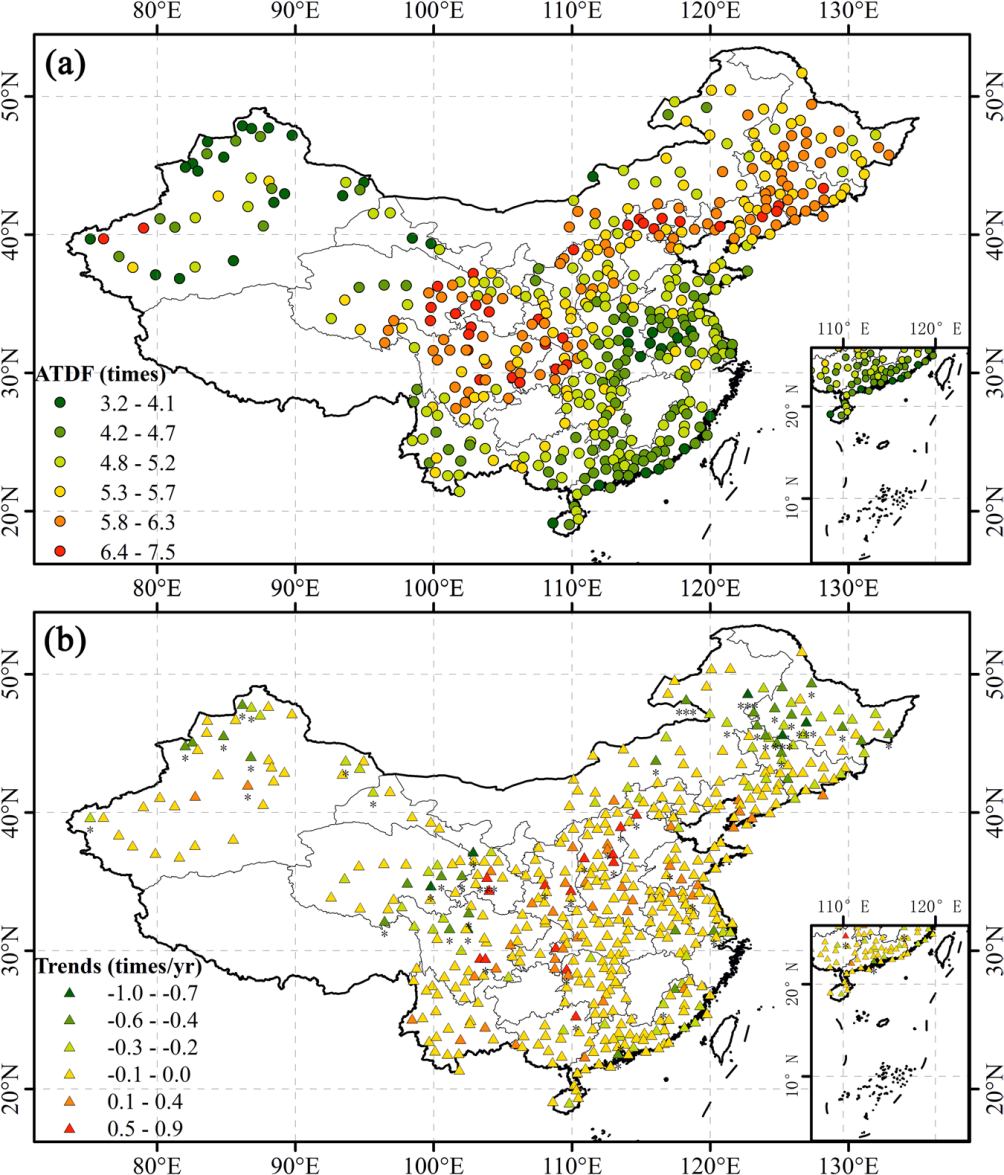
Distributions of (**a**) ATDF in the study area and (**b**) its trends at each station (“***” denotes P-value < 0.001, “**” denotes P-value < 0.01, and “*” denotes P-value < 0.05).

Figure 8 shows that the spatial distribution of ATDF is different from those of ATDS and ATDD. The frequency of drought at some stations in the Xinjiang region of northwest China was not high (i.e., low ATDF values). Stations in the northeastern and southwestern regions had a higher frequency of drought events (i.e., higher ATDF values). Overall, drought events at some stations in the Xinjiang region of northwest China were highly severe and lasted for a long time but did not occur often. Drought events at some stations in the northeastern region were not as severe and did not last a long time. However, they occurred frequently (Fig. 8a). In general, the multi-year trend of ATDF was not significant (Fig. 8b).

### Summary of the data validation

SPI is the most commonly used indicator worldwide for detecting and characterizing droughts because its calculation requires fewer parameters. This index can also better reflect drought intensity and duration at different timescales^61^. SPI has been applied in many fields such as ecology, meteorology, agriculture, water conservancy, among others^62,63^. However, current formulations of SPI cannot monitor droughts below the one-month scale or accurately identify the exact time of drought events. Therefore, based on the commonly used monthly SPI^45^ and the daily SPEI algorithm described in our previous study^48^, we have developed a new daily SPI dataset. The selection of a parameter probability distribution is the key to calculating SPI because an appropriate parameter probability distribution can improve the accuracy of SPI monitoring drought events^43^. We used the gamma probability distribution validated using data from Europe and to be verified over a larger area in the future to calculate the SPI^45^.

To verify the validity of our daily SPI dataset, we selected three typical stations in different regions, i.e., stations 53898 (Henan), 58847 (Fujian), and 56856 (Yunnan), and analyzed the characteristics of drought events at different stations and different timescales. The SPI curve at a longer timescale captured longer-length drought events, mainly because the long-timescale SPI is not sensitive to short-term precipitation. Overall, drought events captured by the new SPI dataset were consistent with those recorded in *The Chinese Disaster Dictionary* and *The Chinese Disaster Yearbook*^57–60^.

SPI at different timescales is closely related to different types of drought (1-month timescale versus meteorological drought, 3- to 6-month timescale versus agricultural drought, 12-month timescale versus hydrological drought, and 24-month timescale versus socioeconomic drought)^22^. We conducted an in-depth analysis of the 3-month-scale SPI widely used in soil and agricultural drought studies. Results show that there are no obvious signs of aggravation of drought in mainland China. Some stations in the northwest, northeast, and southeast regions of China have even shown signs of drought reduction, also reported by others^54,64^. Although there was no obvious drought intensification or mitigation in Hebei, Shanxi, and other places in central China, drought events were nevertheless serious. Note that this region is the food production base of China, so more attention should be paid to drought disasters in this region to avoid serious impacts on agricultural production. According to our 3-month-scale SPI dataset, the characteristics of drought events in northwest Xinjiang were opposite to those in northeast China, also noted by others^54,64^. The causes of different drought events in different regions may be related to location, topography, climate, and other factors^65^. This warrants further analysis and discussion with more data in the future.

In summary, drought has a profound impact on human beings, and modern societies are less inclined to accept the conventional risks of drought. It is thus necessary to make as accurate an assessment and monitoring of drought as possible. The estimation of drought will continue to occupy the attention of ecologists, meteorologists, and hydrologists, among others. The new daily SPI dataset developed and presented here can effectively identify multiple types of droughts and accurately capture the beginning and end of drought events. This dataset will likely be of interest to drought researchers in different fields as well as act as a useful reference for water resources management and drought risk management purposes.

## Data Availability

All calculations of daily SPI are based on the Python language and are available at GitHub: https://github.com/wangqianfeng23/DailySPI. Any updates will also be published on GitHub.
